# Hereditary angioedema with C1 inhibitor deficiency: experience of a new reference center

**DOI:** 10.1186/1939-4551-8-S1-A135

**Published:** 2015-04-08

**Authors:** Sandra Mitie Ueda Palma, Fabiane Milena Castro Araújo Pimenta, Vivian Alves Costa, Ana Karolinne Burlamaqui Melo, Aline Lury Aoki, Neusa Falbo Wandalsen, Anete Grumach, Rosemeire Navickas Constantino-Silva

**Affiliations:** 1 Faculty of Medicine ABC, SP, Brazil

## Background

Hereditary Angioedema (HAE) is an autosomal dominant disorder resulting from a deficiency of C1 esterase inhibitor (C1-INH). It is a rare disease with clinical manifestations debilitating and potentially fatal. The aim of this study was to report the clinical and laboratory characteristics and treatment of patients with Hereditary Angioedema with C1-INH deficit Outpatient Immunology University.

## Methods

This was a retrospective study using data from the clinical records of patients with confirmed HAE with C1-INH diagnosis. The laboratory diagnosis was made after dosages of C4 and C1-INH and functional study of C1-INH (Technoclone ® kit). Age at time of first appointment, onset of symptoms, time to diagnosis, clinical manifestations, prodrome, angioedema triggered the crisis, the need for hospitalization, prophylactic treatment and medication used for seizures were analyzed.

## Results

Were included 30 patients (22F:8M; 16 days of age – 51 years old) diagnosed in the last 2 years. The first symptoms occurred: (6/30; 20%) before 2 years old; (6/30; 20%), most of cases (10/30; 33,3%) ocurred in the adolescence and two patients were asymptomatic. The following clinical manifestations were reported: subcutaneous edema in 86%; 56.6% affecting the face; abdominal pain in 80% and 33.3% of them were submited to abdominal surgery; 46.6% reported asphyxia and 28,5% had voice changing. Prodromal symptoms were referred in 36.6%: cutaneous rash, tingling and pruritus, triggering factors were: trauma (6/30; 53.3 %), stress (17/30; 56.6%) and pregnancy was reported by 4 patients. Hospitalization was referred by 63.3% and out of them, 21% in Intensive Care Unity (ICU). Therapy was employed: danazol (14/30), oxandrolon (13/30) and tranexamic acid (15/30), plasma (4/30). Icatibant was available and applied in 12 patients.

## Conclusions

Clinical manifestations did not differ from the previous reports. It was relevant the high frequency of hospitalization as well as ICU admission. This situation reflects the restricted access to therapy of the attacks. In addition, previous abdominal surgery was also reported in a third of the patients. Although the knowledge about HAE has improved in our country, the access to therapy and management as a whole are still restricted.

* Study supported with a grant from Shire Research Program.

